# Legionnaire’s disease presenting as bilateral central scotomata: a case report

**DOI:** 10.1186/s12879-020-05715-y

**Published:** 2021-01-07

**Authors:** Sho Yamada, Takamasa Kitajima, Satoshi Marumo, Motonari Fukui

**Affiliations:** Respiratory Disease Center, Kitano Hospital, Tazuke Kofukai Medical Research Institute, Osaka, Japan

**Keywords:** Legionnaire’s disease, Bilateral central scotomata, Ocular involvement, Exudative retinal detachment, Case report

## Abstract

**Background:**

Legionnaire’s disease is one of the major causes of community-acquired pneumonia and is occasionally complicated by neurological symptoms. However, reports of ocular lesions due to Legionnaire’s disease are limited.

**Case presentation:**

We report the case of a patient with Legionnaire’s disease presenting as bilateral central scotomata due to retinal lesions. The patient consulted due to fever and bilateral central scotomata, as well as other extrapulmonary symptoms. Optical coherence tomography (OCT) showed bilateral accumulations of fluid under the retina, and the patient was diagnosed with bilateral exudative retinal detachment. Later, Legionnaire’s disease was confirmed by pulmonary infiltrates on chest imaging and positive urinary antigen for *Legionella pneumophila*. After administration of antibiotics, the bilateral central scotomata and bilateral subretinal fluid accumulations completely resolved, as did the other extrapulmonary symptoms and the pulmonary infiltrates. Thus, the bilateral central scotomata due to exudative retinal detachment were thought to be caused by Legionnaire’s disease.

**Conclusions:**

This case demonstrates that Legionnaire’s disease can present as bilateral central scotomata. We may consider the possibility of extrapulmonary involvement complicating Legionnaire’s disease when we encounter bilateral ocular lesions in patients with fever and pneumonia.

## Background

*Legionella pneumophila* pneumonia accounts for approximately 1.7–5.4% of hospitalizations for community-acquired pneumonia, and is associated with high rates of admission to the intensive care unit and high in-hospital mortality [1, 2]. One of the distinct features of Legionnaire’s disease is its wide range of extrapulmonary manifestations, especially its neurological symptoms such as headache, altered mental status, hallucinations, ataxia, and other focal symptoms [3, 4]. However, there are a limited number of reports on ocular involvement, and particularly on retinal lesions [5, 6], in Legionnaire’s disease. We herein report a patient with Legionnaire’s disease presenting with bilateral central scotomata due to exudative retinal detachment.

## Case presentation

**Fig. 1:**
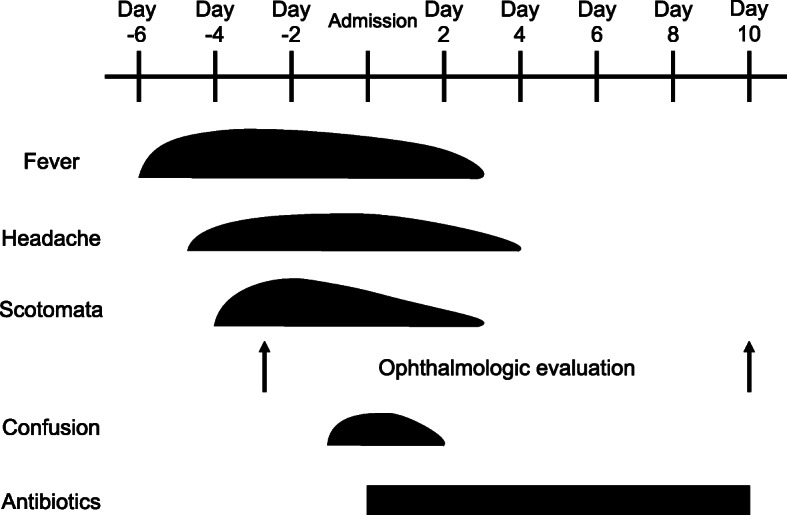
Clinical course of the patient

**Fig. 2:**
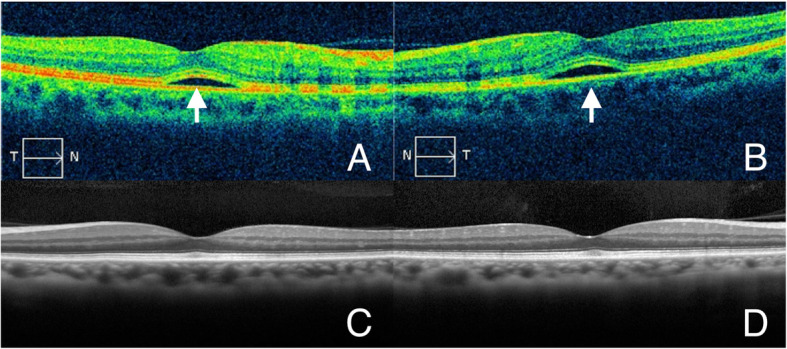
Optical coherence tomography of both eyes before admission (**a**: right, **b**: left) and on day 10 (**c**: right, **d**: left). (**a**, **b**) Bilateral subretinal fluids (white arrows) under the macula. (**c**, **d**) Absence of bilateral subretinal fluids under the macula

A 64-year-old man with an unremarkable medical history was admitted to our hospital due to fever and scotoma. Six days before admission, he developed fever and a non-productive cough. Two days later, he became aware of a black spot in the center of his visual field. He visited an ophthalmologist, and his symptoms were interpreted as bilateral central scotomata (Fig. 1). He was diagnosed with bilateral exudative retinal detachment on optical coherence tomography (OCT) (Fig. 2a, b). Fundus examination was normal. The cause of the fever and bilateral central scotomata was unclear, and he was referred to our hospital for further neurological evaluation.

On admission, he had a variety of symptoms including fever, mild headache, confusion, diarrhoea, urinary incontinence, and mild non-productive cough. The central scotomata improved, but he still complained of a round yellowish lesion in the bilateral central visual fields. He had a temperature of 38.7 °C, blood pressure of 108/78 mmHg, pulse rate of 100/min, and respiratory rate of 30/min, with an oxygen saturation of 93% on room air. His breath sounds were diminished in the right lower lung field. He showed disorientation to place, but the rest of the neurological examinations were normal.

Laboratory tests revealed leucocytosis with neutrophilia. C-reactive protein (CRP) and procalcitonin levels were elevated. Blood chemistry examinations showed hyponatremia, renal insufficiency, elevated serum aminotransferases, and high lactic dehydrogenase (LDH) levels (Table 1). Urinalysis showed microscopic haematuria (urine occult blood 2+, urine red blood cells 1–4/high power field), and proteinuria (urinary protein 2+, spot urine protein/ creatinine ratio of 1.20 g/g creatinine) without leucocyturia.

**Table 1:** Laboratory findings on admission

Test	Results	Reference range
White blood cell count	12.8 (10^9^/L)	3.3–8.6
Neutrophil ratio	93.8 (%)	41.7–73.7
C-reactive protein (CRP)	31.91 (mg/dL)	0.0–0.14
Procalcitonin	1.30 (ng/dL)	0.0–0.10
Serum sodium	131 (mmol/L)	138–145
Serum creatinine	1.12 (mg/dL)	0.65–1.07
Serum aspartate aminotransferase (AST)	152 (U/L)	13–30
Serum alanine aminotransferase (ALT)	222 (U/L)	10–42
Serum lactate dehydrogenase (LDH)	479 (U/L)	124–222

This table shows the results of the patient’s laboratory tests on admission

We suspected meningoencephalitis based on several symptoms, including the visual abnormality, fever, headache, and confusion. Lumbar puncture and head imaging were performed following the initial examination. Cerebrospinal fluid analysis revealed a mildly elevated protein level of 55.7 mg/dL, a normal cell count, and a normal glucose level of 92 mg/dL. Brain magnetic resonance imaging did not reveal any mass lesions, inflammatory processes, or acute ischemic changes. Thus, the possibility of brain tumours, meningitis, encephalitis, and stroke could be excluded.

**Fig. 3:**
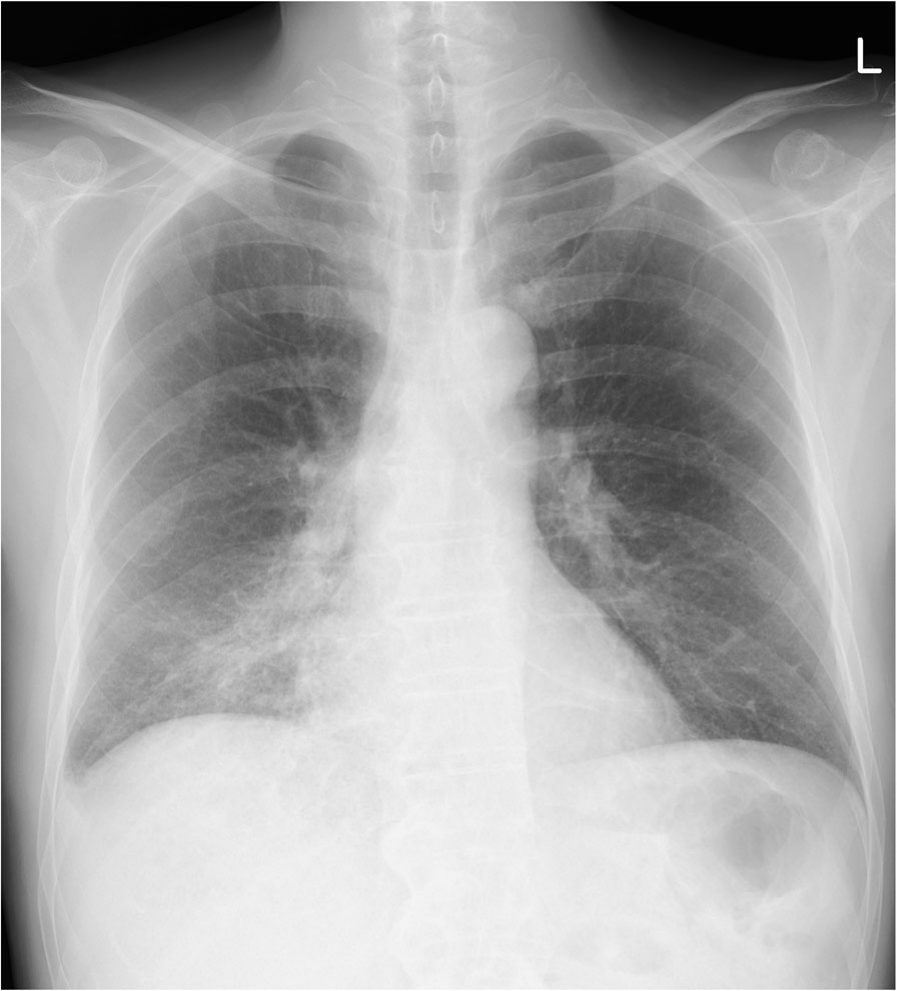
Chest radiograph on admission showing infiltrates in the right lower lung field

**Fig. 4:**
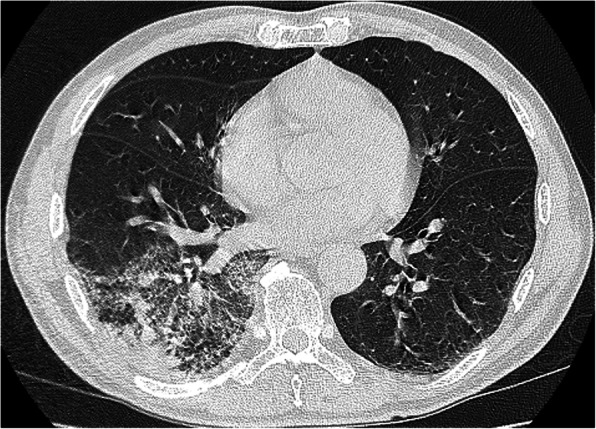
Chest computed tomography on admission showing consolidation in the right lower lobe

Chest radiography, which was performed to determine a possible fever origin, showed infiltrates in the right lower lung field (Fig. 3). Chest computed tomography revealed consolidation in the right lower lobe (Fig. 4). To detect the aetiology of lobar pneumonia, the urinary antigen test (Binax NOW, Alere, Orlando, FL, USA) was performed that was positive for serogroup 1 of *Legionella pneumophila*. Therefore, a diagnosis of Legionnaire’s disease was made. He did not receive previous antibiotic therapy for the present illness and treatment with levofloxacin and azithromycin was initiated on the day of admission after the diagnosis. After antibiotics were administered, the patient’s symptoms and laboratory abnormalities simultaneously normalized. The visual symptoms disappeared on day 3. An ophthalmologic examination, including OCT, was done on day 10 which revealed complete resolution of the bilateral retinal lesions (Fig. 2c, d). The patient was discharged, and the antibiotics were continued until completion at 10 days of administration. To reveal the possible source of infection, samples from an old water dispenser used by the patient at his home were collected for Legionella culture. Although Legionella species were not detected, the patient was advised not to use that dispenser. The local health care centre conducted epidemiological surveillance; however, an outbreak was not reported.

## Discussion and conclusions

We report a rare case of Legionnaire’s disease complicated by extrapulmonary signs and symptoms, including bilateral central scotomata due to exudative retinal detachment.

We confirmed *Legionella pneumophila* pneumonia by chest imaging and urinary antigen testing. Legionnaire’s disease is known to be associated with several characteristic extrapulmonary symptoms and clinical findings. Several clinical scores incorporate clinical and laboratory findings to predict *Legionella pneumophila* pneumonia [3, 7–10]. Among the previously reported predictors, headache, mild confusion, diarrhoea, absence of sputum, hyponatremia, remarkably elevated serum levels of CRP and LDH, and microscopic haematuria were present in the current case. Bilateral central scotomata developed simultaneously with these symptoms and the laboratory findings, and disappeared altogether after the administration of antimicrobial agents. These findings suggest that bilateral central scotomata should also be considered an extrapulmonary symptom of Legionnaire’s disease in this patient.

Although we found two previous cases reporting retinal involvement in Legionnaire’s disease [5, 6], neither had retinal detachment. One case did not have pneumonia, and endocarditis might have been responsible for the ocular findings [5]. The other was caused by *Legionnaire longbeachae 1,* and ophthalmologic symptoms occurred 13 days after the onset of pulmonary symptoms and did not completely resolve within 6 months [6]. In addition, both cases were diagnosed based only on increased serum antibody titres. Thus, the causative association between Legionnaire’s disease and the ocular lesions was not clear in these cases, but that was evident in our case. To the best of our knowledge, this is the first reported case of Legionnaire’s disease complicated by bilateral central scotomata.

The differential diagnosis for binocular visual field loss includes various diseases that affect any component of the visual pathway from the eye to the cortex [11]. However, the bilateral distribution of the visual symptoms in this patient urged us to perform neuroimaging, as this distribution can be seen in neurological emergencies such as bilateral optic nerve abnormality, compression of the optic chiasm, and stroke [12]. Additionally, OCT revealed bilateral exudative retinal detachment, which was consistent with bilateral central scotomata. Retinal lesions might not have been recognized if ophthalmologic evaluation had not been performed. In our case, OCT was of notable value because, unlike in previous cases describing fundal abnormalities [5, 6], no apparent lesion was detected in the fundus examination of our patient.

The lesion corresponding to the bilateral central scotomata in this patient was exudative retinal detachment. Exudative retinal detachment occurs when the integrity of the blood-retinal barrier is damaged and fluid accumulates under the retina. This is caused by several disease processes, including inflammatory, infectious, malignant, vascular, and degenerative conditions [13]. The prevalence of exudative retinal detachment in patients with ocular inflammatory diseases is approximately 1 % [14]. Exudative retinal detachment associated with systemic infection has been reported mainly in case reports or case series and the exact occurrence rate is unknown. Among infectious diseases, tuberculosis, syphilis, Lyme disease, cat-scratch disease, systemic fungal infections, and viral agents can cause exudative retinal detachment [13, 15]. However, exudative retinal detachment due to Legionnaire’s disease has never been reported and we believe that some of these occurrences might remain unrecognized. In infectious diseases, it is thought to be caused by the direct microbial invasion or indirectly by immune-mediated responses. Since the retinal lesions in our case existed equally in both eyes, it was likely that an immune-mediated response was involved in the pathogenesis. These retinal lesions promptly improved after antibiotic administration. Previous reports have suggested the efficacy and safety of systemic corticosteroids when exudative retinal detachment did not respond to antibiotic treatment [13]. The immune-mediated nature of the underlying process might also be supported by the renal abnormalities. Renal pathology of the Legionnaire’s disease includes acute tubular necrosis, tubulointerstitial nephritis, and glomerulonephritis [16]. Reduced renal function, microscopic haematuria, and proteinuria in this patient suggested the presence of tubulointerstitial nephritis, glomerulonephritis, or both. The circulating endotoxin might have contributed to the renal manifestation [17], and we speculate that a similar pathogenesis could be involved in the retinal lesion.

In conclusion, this case highlighted a clinically important issue: namely, that Legionnaire’s disease can present as bilateral central scotomata, and ophthalmologic examinations together with neurological imaging play an important role in detecting the corresponding lesions. Physicians may consider Legionnaire’s disease when they see patients with fever, pneumonia, and ocular symptoms, and should perform ophthalmologic evaluations.

## Data Availability

Not applicable.

## Abbreviations

ALT: Alanine aminotransferase
AST: Aspartate aminotransferase
CRP: C-reactive protein
LDH: Lactic dehydrogenase
OCT: Optical coherence tomography
